# Evaluation of Biventricular Diastolic Function in Preterm Infants in the First Week of Postnatal Life

**DOI:** 10.1007/s00246-025-03974-7

**Published:** 2025-07-29

**Authors:** Ivonne Sierra-Strum, Rutuja Kibe, Piyawat Arichai, Hemananda Muniraman, Ebrahimi Mahmood, Rowena Cayabyab, Yogen Singh, Rangasamy Ramanathan

**Affiliations:** 1 Department of Pediatrics, Division of Neonatal Medicine, Keck School of Medicine, Los Angeles General Medical Center, University of Southern California, 2051 Marengo Street, IRD Building, Room 820, Los Angeles, CA 90033 USA; 2 Department of Pediatrics, Division of Neonatology, Kapiolani Medical Center for Women and Children, Honolulu, HI USA; 3 Department of Pediatrics, Division of Neonatology, Cedars Sinai Guerin Children’s, Cedars Sinai Medical Center, Los Angeles, CA USA; 4 Department of Pediatrics, Division of Neonatology, UC Davis Children’s Hospital, UC Davis Health, Sacramento, CA USA

**Keywords:** Preterm infants, Echocardiography, Diastolic function, Tissue doppler imaging, Left atrium

## Abstract

Diastolic dysfunction contributes significantly to respiratory instability in preterm infants but remains under-characterized during early postnatal transition. We aimed to evaluate the evolution of biventricular diastolic function during the first week of life in very low birth weight (VLBW) preterm infants. In this prospective observational study, 20 preterm infants (< 32 weeks gestation or < 1500 g) underwent serial echocardiograms at 24, 48, 72 h, and day 7. TDI mitral/tricuspid inflow velocities, left atrial (LA) volume, cardiac output, and shunt characteristics were measured. Hemodynamically significant PDA (hsPDA) was defined by standard echocardiographic criteria. Mitral E/e′ decreased from 16.4 [14.2–18.3] on day 1 to 13.1 [11.3–15.1] by day 7 (*p* < 0.01), while lateral e′ increased from 3.3 [2.9–3.7] to 4.1 [3.6–4.6] cm/s (*p* = 0.009), consistent with improving relaxation. At day 7, infants with hsPDA (6/20) had higher mitral E/A ratios (1.08 [1.01–1.14] vs. 0.95 [0.89–1.00], *p* = 0.04), elevated E/e′ (9.1 [8.4–10.0] vs. 6.4 [5.7–7.1], *p* < 0.01). Indexed LA volume was also larger in the hsPDA group. Larger PFO diameter (≥ 2.5 mm) showed a trend toward lower LA volume indices and E/e′, although differences were not statistically significant. Rapid diastolic adaptation occurs during the first postnatal week in VLBW infants. hsPDA is linked to increased LA pressure and impaired relaxation. Early functional echocardiography may aid in guiding targeted neonatal care.

**Supplementary Information:**

The online version contains supplementary material available at 10.1007/s00246-025-03974-7.

## Introduction

Cardiorespiratory instability remains a significant challenge in the early care of premature infants. These neonates must rapidly adapt to significant shifts in preload and afterload, despite having an immature myocardium that may be limited in its ability to sustain adequate cardiac output [1].

A central aspect of this cardiovascular adaptation is diastolic function: the heart’s capacity to relax and accommodate venous return. In preterm infants, diastolic function is frequently compromised due to immature contractile structures, underdeveloped sarcoplasmic reticulum less efficient at calcium-handling systems, and reduced compliance related to disorganized and stiff collagen fibers [2]. These limitations can impair ventricular filling, elevate pulmonary venous pressure, and contribute to respiratory distress during this vulnerable period.

Left ventricular diastolic dysfunction has been shown to be relatively common in extremely preterm infants under 32 weeks of gestation, often influenced by prolonged exposure to increased volume load [3]. During the first days to weeks of life, diastolic function evolves, with parameters such as trans mitral flow velocities and tissue Doppler imaging (TDI) indices demonstrating nonlinear improvement. These changes reflect the gradual maturation of ventricular relaxation and compliance as the neonatal myocardium adapts to extrauterine life [4].

Diastolic dysfunction has been increasingly recognized as a contributor to adverse respiratory outcomes in neonates [5, 6]. Elevated E/e′ ratios, which serve as a noninvasive surrogate for increased left ventricular filling pressures, have been associated with respiratory compromise in preterm infants, as demonstrated by de Waal et al. [7]. Furthermore, Bussmann et al. identified left ventricular diastolic dysfunction as an independent predictor of the need for mechanical ventilation in this population [8].

Our cohort comprised of very low birth weight (VLBW) infants who mainly received noninvasive respiratory support throughout their first postnatal week. This allowed us to explore diastolic function under distinct loading conditions, given noninvasive modalities tend to impose less intrathoracic pressure variation and may thus have less impact on altering ventricular filling dynamics [9]. While prior studies have included preterm infants managed with noninvasive modalities [10], prospective longitudinal evaluations during the immediate transitional period remain limited, particularly in noninvasively ventilated VLBW infants.

In this context, a thorough appraisal of diastolic performance during the transitional circulation is critical. Defining normative maturation trajectories and detecting early departures may facilitate risk stratification and guide targeted interventions. The aim of our study was to characterize the evolution of biventricular diastolic function in VLBW preterm infants over their first week of life.

## Methods

### Study Design and Population

This prospective observational study was conducted between May 2023 and February 2024 in the Level III neonatal intensive care unit (NICU) at Los Angeles General Medical Center. Eligible participants included inborn preterm infants with a birth weight < 1500 g or gestational age < 32 weeks who were clinically stable to undergo scheduled echocardiographic evaluation. Infants were enrolled antenatally when feasible or within the first 12 h of life. Exclusion criteria included major congenital heart disease (excluding patent foramen ovale or patent ductus arteriosus), significant structural anomalies, or known genetic syndromes. Written informed consent was obtained from a parent or legal guardian prior to enrollment. The study was approved by the Institutional Review Board (IRB) of the University of Southern California (Proposal #HS-23–00184) on April 25, 2023. The protocol was determined to involve no greater than minimal risk and qualified for expedited review in accordance with 45 CFR 46.110. Data collection and study procedures were conducted in accordance with approved protocols and HIPAA regulations.

### Echocardiographic Protocol and Measurements

Serial transthoracic echocardiograms were performed at approximately 24, 48, and 72 h, and on day 7 of life to evaluate cardiovascular transition over the first postnatal week. Imaging was acquired at the bedside using a GE E90 ultrasound system (GE Healthcare, IL, USA) with a 12 MHz neonatal probe. Acquisition was ECG-gated and performed according to the guidelines of the American Society of Echocardiography (ASE) [11]. Echocardiograms were obtained by trained neonatal-perinatal medicine fellows and subsequently reviewed by a pediatric cardiologist. To assess reproducibility, intra-observer variability was evaluated in a subset of studies (~ 73%) by repeating key measurements across at least two cardiac cycles. All measurements were performed and reviewed by a single investigator (I.S.) to minimize inter-observer variability. All studies were stored digitally and analyzed offline using Cerner SkyVue (Cerner Corporation, Kansas City, MO, USA).

Cardiac measurements included two-dimensional, M-mode, pulsed-wave Doppler, and TDI. From the apical four-chamber view, mitral and tricuspid inflow velocities were *obtained using pulsed-wave Doppler with the sample volume positioned at the tips of* the atrioventricular valve leaflets. Early (E) and late (A) diastolic peak velocities were measured, and the E/A ratio was calculated for each valve.

TDI was used to assess annular motion. The sample volume was placed at the septal and lateral mitral annulus and the lateral tricuspid annulus to measure peak systolic (s′), early diastolic (e′), and late diastolic (a′) velocities. The E/e′ ratio was calculated by dividing the mitral E velocity by the average of septal and lateral e′ velocity to estimate diastolic relaxation patterns. Atrial filling fraction was calculated using pulsed-wave Doppler tracings from the apical four-chamber view. The AFF was defined as the ratio of A wave velocity time integral (A-VTI) to the sum of E and A VTIs: AFF = A-VTI/(E-VTI + A-VTI)**,** expressed as a decimal. This measurement reflects the relative contribution of atrial contraction to ventricular filling and was obtained from mitral inflow Doppler recordings over three cardiac cycles.

Left atrial volume was assessed in the apical four-chamber view using the method of disks, excluding the pulmonary veins and left atrial appendage. While the American Society of Echocardiography (ASE) recommends indexing left atrial volume to body surface area (BSA), we indexed to body weight (mL/kg) to better align with the physiologic relevance in preterm infants, where BSA calculations may be less precise and weight-based indexing provides more practical and consistent comparisons. Notably, prior studies including those by Toyoshima et al., have also used weight-based indexing and demonstrated its utility in assessing PDA severity and predicting the need for surgical intervention [12, 13]. These findings support our methodological approach in this population.

Left and right ventricular outputs were measured using pulsed Doppler-derived velocity time integrals (VTI) at the level of the left ventricular outflow tract (LVOT) and pulmonary valve, respectively, in combination with respective valve annulus diameter to estimate stroke volume and cardiac output.

The ductus arteriosus was assessed in the high left parasternal short-axis view. Measurements included the ductal diameter at end-systole and the direction of flow (left-to-right, right-to-left, or bidirectional) using color and spectral Doppler. The left atrium-to-aortic root ratio (LA: Ao) was measured in the parasternal long-axis view. Hemodynamically significant PDA was defined by at least two of the following echocardiographic criteria: ductal diameter > 1.5 mm, LA: Ao ≥ 1.4, diastolic flow reversal in the descending aorta, and left pulmonary artery (LPA) end-diastolic velocity ≥ 20 cm/sec. The presence and direction of shunting through a patent foramen ovale were recorded using color Doppler from subcostal views.

In cases where E and A waves were fused due to elevated heart rate or technical limitations, infants were soothed using facilitated tucking and oral sucrose when feasible. Studies with persistent diastolic wave fusion were excluded from final analysis for Doppler-derived E and A velocities and E/A ratios.

### Clinical Data Collection

Clinical and demographic data were collected for all enrolled infants. Perinatal variables included gestational age, birth weight, sex, Apgar scores at 1, 5, and 10 min, mode of delivery, small for gestational age (SGA) status, antenatal steroid exposure, and maternal ethnicity and race.

At each echocardiographic time point, we recorded mode of respiratory support, fraction of inspired oxygen (FiO_2_) and blood pressure. Additional clinical data collected during the first postnatal week included the presence of hypotension requiring treatment, intraventricular hemorrhage (IVH), suspected or culture-positive sepsis, and need for mechanical ventilation.

Given that three infants received inotropic medications concurrent with echocardiographic assessment, we performed a sensitivity analysis excluding all echocardiograms obtained during inotrope exposure.

Management of the hsPDA followed unit-based clinical protocols or at the discretion of neonatal provider and was not guided by the research echocardiographic findings.

### Statistical Analysis

This study was descriptive in nature and aimed to characterize the trajectory of echocardiographic measurements over time. Continuous variables were assessed for normality using the Shapiro–Wilk test; however, due to the small sample size, most continuous variables are reported as median with interquartile range (IQR) to provide a more robust summary of central tendency. Categorical variables are summarized as counts and percentages. Given the small sample size and non-normal distribution of most variables, all temporal comparisons of echocardiographic indices were performed using the Friedman test for non-parametric repeated measures. Between-group comparisons were made using the Mann–Whitney U test. Sensitivity analyses were conducted to compare primary findings with and without data obtained during inotropic infusion**,** assessing consistency in direction and magnitude of diastolic function trends. All statistical analyses were conducted using SPSS version 27 (IBM Corp., Armonk, NY) and GraphPad Prism version 9 (GraphPad Software, San Diego, CA). A two-tailed *p*-value < 0.05 was considered statistically significant.

## Results

### Patient Characteristics

A total of 20 preterm infants were enrolled. The median gestational age at birth was 28.0 weeks [IQR 26.8–29.4], and the median birth weight was 930 g [IQR 810–1080]**.** Six infants (30%) were classified as small for gestational age (SGA). The cohort included 11 male (55%) and 9 female (45%) infants. The median Apgar scores were 5 [IQR 4–6] at 1 min and 7 [IQR 6–8] at 5 min. Most infants (70%) were of Hispanic ethnicity. Delivery was by cesarean section in 15 of 20 infants (75%). Antenatal corticosteroids were administered to 17 mothers (85%). At the time of the initial echocardiogram, 3 infants (15%) were receiving invasive mechanical ventilation, while the remaining 17 (85%) were on noninvasive respiratory support. By day 7, 85% of the cohort remained on noninvasive support. Clinical morbidities included treated hypotension in 3 infants (15%) and intraventricular hemorrhage in 7 infants (35%), of which two cases (10%) had grade III or IV intraventricular hemorrhage. Notably, two infants received Dopamine (2.5–10 µg/kg/min) during the 48 h echocardiogram, and a third infant was on Dopamine infusion (5–10 µg/kg/min) at the day 7 echo timepoint. Additional demographic and perinatal characteristics are summarized in Table 1.

**Table 1:** Demographics and Clinical Characteristics Demographics and Clinical Characteristics of Study Population presented as median [interquartile range] or n (%)

Variable	Value (Median [IQR]/n (%))
Number of infants	20
Gestational age at birth (weeks)	28 [26.8–29.4]
Birth weight (g)	930 g [810–1080]
Male sex (%)	11 (55%)
Small for gestational age (%)	6 (30%)
Antenatal steroids (%)	17 (85%)
Apgar score at 1 min (median [IQR])	5 [4–6]
Apgar score at 5 min (median [IQR])	7 [6–8]
Invasive ventilation during first week (%)	3 (15%)
hsPDA at 48 h (%)	7 (35%)
hsPDA at 7 days (%)	6 (30%)
Pharmacologic PDA treatment at day 7 (%)	4 (67% of hsPDA group)^*^

hsPDA hemodynamically significant Patent Ductus Arteriosus

^*^Proportion calculated within the subgroup of infants diagnosed with hsPDA at day 7

### Longitudinal Cardiac Function Trends

**Fig. 1:**
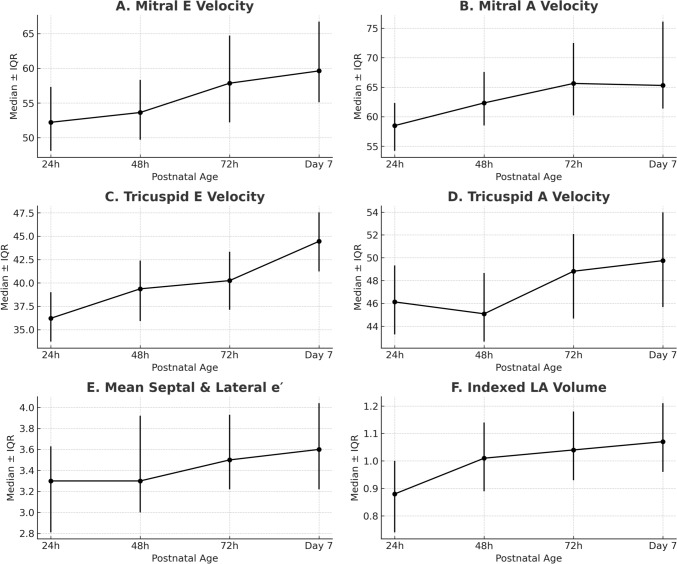
Longitudinal changes in diastolic function and left atrial indices in preterm infants during the first week of life Mitral inflow E/A ratio (**a**), lateral mitral annular e′ velocity (**b**), mitral E/e′ ratio (**c**), and indexed left atrial volume (**d**) are shown from 24 h to day 7. Median values and interquartile ranges are depicted. *N* = 20 infants

Serial echocardiographic assessments demonstrated evolving diastolic function during the first week of life (Table 2; Fig. 1). At 24 h of life, the mitral E/A ratio was < 1.0 in most infants (median 0.90 [IQR 0.82–0.98]). The ratio decreased slightly at 48 h (median 0.86 [IQR 0.79–0.93]), followed by a modest increase at 72 h (median 0.89 [IQR 0.82–0.96]) and at day 7 (0.92 [IQR 0.85–0.99]). These changes were not statistically significant (*p* = 0.09, Friedman test). E and A wave fusion precluded discrete measurement in 3 infants (15%) during early time points.

**Table 2:** Diastolic function Trends over Time Presented as Median [Interquartile Range]

Parameter	24 H	48 H	72 H	Day 7
Mitral E (cm/s)	52.22 [48.10–57.33]	53.63 [49.71–58.32]	57.85 [52.2–64.72]	59.62 [55.1–66.71]
Mitral A (cm/s)	58.50 [54.21–62.33]	62.34 [58.52–67.57]	65.64 [60.23–72.51]	65.30 [61.37–76.10]
Mitral E/A Ratio	0.90 [0.82–0.98]	0.86 [0.79–0.93]	0.89 [0.82–0.96]	0.92 [0.85–0.99]
Mean Septal and Lateral e′ (cm/s)	3.3 [2.810–3.63]	3.3 [3.00–3.92]	3.5 [3.22–3.93]	3.6 [3.22–4.04]
Mitral E/e′ Ratio	16.4 [14.23–18.31]	13.7 [11.80–15.52]	14.2 [12.01–16.12]	13.1 [11.33–15.14]
Tricuspid E (cm/s)	36.22 [33.72–39.01]	39.37 [35.92–42.39]	40.25 [37.12–43.34]	44.45 [41.23–47.54]
Tricuspid A (cm/s)	46.13 [43.28–49.32]	45.09 [42.65–48.67]	48.82 [44.67–52.07]	49.75 [45.67–53.97]
Tricuspid E/A Ratio	0.78 [0.69–0.86]	0.86 [0.78–0.94]	0.83 [0.74–0.92]	0.89 [0.81–0.97]
Tricuspid e′ (cm/s)	4.6 [4.13–5.13]	4.8 [4.33–5.42]	5.0 [4.51–5.62]	5.1 [4.62–5.73]
Indexed LA Volume (mL/kg)	0.88 [0.74–1.00]	1.01 [0.89–1.14]	1.04 [0.93–1.18]	1.07 [0.96–1.21]
Atrial Filling Fraction	0.55 [0.50–0.62]	0.52 [0.47–0.58]	0.51 [0.46–0.57]	0.50 [0.44–0.56]
LV Output (mL/kg/min)	191 [178–210]	281 [260–306]	280 [258–305]	278 [252–310]
RV Output (mL/kg/min)	205 [190–226]	265 [240–288]	264 [238–287]	263 [235–290]

*LA* left atrium, *E* early diastolic velocity, *A* late diastolic velocity, *e′* TDI early diastolic velocity; *LV* left ventricular, *RV* right ventricular, *mL/kg* milliliters per kilogram, *mL/kg/min* milliliters per kilogram per minute

The average mitral annular e′ velocity, calculated as the mean of septal and lateral e′, increased from 3.3 cm/s [IQR 2.9–3.6] at 24 h to 3.9 cm/s [IQR 3.5–4.3] at day 7 (*p* = 0.004).

Mitral E/e′ ratio decreased over time, from 16.4 [IQR 14.2–18.3] to 13.1 [IQR 11.3–15.1] by day 7 (*p* < 0.01). The most pronounced reduction occurred between 24 and 48 h. In three infants who required invasive mechanical ventilation during the first week, E/e′ remained elevated (> 16) at day 7 despite overall improvement in the cohort.

Indexed left atrial (LA) volume increased from 0.88 mL/kg [IQR 0.74–1.00] at 24 h to 1.07 mL/kg [IQR 0.96–1.21] at day 7 (*p* = 0.12). When stratified by gestational age, infants < 27 weeks (*n* = 9) showed a more pronounced increase (median change + 0.29 mL/kg, IQR 0.18–0.41) compared to those ≥ 27 weeks (+ 0.12 mL/kg, IQR 0.05–0.19). Atrial filling fraction followed a similar trend, with a median of 0.55 [IQR 0.50–0.62] at 24 h and 0.50 [IQR 0.44–0.56] at day 7.

Right-sided parameters were tracked in all infants. Tricuspid E/A ratio increased from 0.78 [IQR 0.69–0.86] to 0.89 [IQR 0.81–0.97] (*p* = 0.02). Tricuspid e′ increased from 4.6 cm/s [IQR 4.1–5.1] to 5.1 cm/s [IQR 4.6–5.7] (*p* = 0.03), though values were highly variable between infants (range at day 7: 3.7–6.3 cm/s).

Sensitivity analysis excluding timepoints with concurrent inotropic support (*n* = 3 timepoints from 3 infants) yielded slightly lower mitral E/e′ values at 48 h (12.8 [IQR 11.0–14.6] vs. 13.7 [11.8–15.5]) and day 7 (12.6 [IQR 10.9–14.0] vs. 13.1 [11.3–15.1]). The average e′ velocity also demonstrated a more pronounced increase in the sensitivity cohort, from 3.3 to 3.9 cm/s (*p* = 0.005). Indexed LA volumes remained within a comparable range, although slightly lower at 48 h (0.97 [0.85–1.07] mL/kg). Overall trends remained directionally consistent (See Supplemental Table S2 for details of sensitivity analysis).

### Impact of Hemodynamically Significant PDA

At 48 h of life, 7 infants (35%) met criteria for a hsPDA, and 6 infants (30%) continued to meet criteria at day 7.

By day 7, infants with hsPDA demonstrated higher mitral E/A ratios (1.08 [IQR 1.01–1.14]) compared to those without PDA (0.95 [IQR 0.89–1.00], *p* = 0.04). Median mitral E/e′ was significantly higher in the hsPDA group (9.1 [IQR 8.4–10.0]) than in non-hsPDA infants (6.4 [5.7–7.1], *p* < 0.01), though overlap between groups was noted. Indexed LA volume was also larger in the hsPDA group (1.14 [IQR 1.05–1.22] mL/kg vs. 0.91 [0.84–0.98] mL/kg, *p* < 0.01), with 3 infants exceeding 1.3 mL/kg at day 7.

Atrial filling fraction was modestly higher in the hsPDA group (median 0.58 [0.51–0.63]). Lateral e′ values trended lower (3.2 [2.9–3.6] vs. 3.7 [3.3–4.0], *p* = 0.06), though distributions overlapped. Notably, none of the non-hsPDA infants had an E/e′ > 10 or LA volume index > 1.2 mL/kg.

At day 7, four of the hsPDA infants (67%) were receiving pharmacologic treatment with acetaminophen, while two had not yet initiated or completed therapy. In the treated infants, LA volume and E/e′ values remained elevated but showed a downward trend compared to untreated hsPDA infants.

Sensitivity analysis excluding echocardiographic timepoints during inotropic exposure (*n* = 3 infants) revealed consistent trends. Among infants with hsPDA, median E/e′ decreased slightly to 8.6 [IQR 7.9–9.7], and indexed LA volume decreased to 1.09 [IQR 1.01–1.19] mL/kg. Although absolute values were lower, both measures remained significantly higher compared to non-hsPDA infants (E/e′: 6.3 [5.7–7.1], LA volume: 0.91 [0.84–0.98] mL/kg, both *p* < 0.01). The mitral E/A ratio and atrial filling fraction were unchanged in sensitivity analysis.

### Cardiac Output Trends

Left ventricular cardiac output increased from 191 [178–210] at 24 h of age to 281 [260–306] mL/kg/min at 48 h of age. By day 7, LVO plateaued at a median of 278 mL/kg/min [IQR 252–310]. Right ventricular output increased from 205 [IQR 190–226] at 24 h to 265 [IQR 240–288] mL/kg/min by 48 h, remaining stable at a median of 263 mL/kg/min [IQR 235–290] through day 7.

Infants with hsPDA demonstrated higher peak LVO values at 48 h.

(301 [IQR 276–326]) compared to those without hsPDA (265 [IQR 234–296] ml/kg/min, *p* = 0.02).

Sensitivity analysis excluding echocardiograms performed during inotropic support demonstrated similar trends in cardiac output. Median LVO at 48 h was 289 [IQR 258–305] mL/kg/min and RV output was 262 [IQR 235–284] mL/kg/min, closely mirroring the original values.

### Exploratory Analysis of PFO Characteristics

A total of 17 infants (85%) demonstrated left-to-right shunting across a patent foramen ovale (PFO) during the first week of life. Of these, 6 infants (35%) had a PFO diameter ≥ 2.5 mm. Stratified analysis by hsPDA status revealed that among infants without hsPDA during the first week of life (*n* = 13), those with a larger PFO (≥ 2.5 mm, *n* = 4) had lower indexed left atrial (LA) volumes (0.89 [IQR 0.83–0.96] mL/kg) and E/e′ ratios (6.1 [5.4–6.7]) compared to those with a smaller PFO (< 2.5 mm, *n* = 9; LA volume: 1.03 [0.94–1.14] mL/kg; E/e′: 7.0 [6.2–8.1]). Among infants with hsPDA during the first week of life (*n* = 7), no consistent association between PFO size and diastolic parameters was observed. No significant differences were observed in mitral E/A ratio or e′ velocities based on PFO size.

## Discussion

Diastolic function refers to the heart’s ability to relax and fill during the cardiac cycle and is governed by the interaction between left atrial (LA) pressure, left ventricular (LV) compliance, and myocardial relaxation. When the mitral valve opens, blood enters the LV driven initially by suction generated from the elastic recoil of myocardial fibers returning toward their resting length. This phase is characterized by a drop in LV pressure, facilitating early rapid filling. As filling continues and the LV cavity expands beyond resting dimensions, increasing myocardial stiffness slows the filling rate and raises LV pressure. Thus, effective diastolic function depends on a balance between relaxation and compliance, both of which are developmentally regulated in preterm infants [1, 2, 14].

In this prospective study of VLBW preterm infants, we observed evidence of diastolic functional maturation over the first week of life. Key echocardiographic indices, such as the mitral E/A ratio, TDI e′ velocity, and E/e′, demonstrated trends consistent with improving left ventricular relaxation and declining filling pressures over the first week of life.

These findings align with previous studies describing early adaptation of diastolic function during neonatal transition. Sirc et al. reported dynamic changes in ventricular filling parameters within the first 48 h in infants < 1250 g, suggesting transient diastolic dysfunction in the immediate postnatal period [15]. Similarly, Kozák-Bárány et al. documented progressive improvement in LV diastolic indices over the first month of life, approaching values typical of term neonates by four weeks [16]. Their findings reinforce the concept that diastolic performance in preterm infants evolves rapidly as the myocardium transitions from fetal to neonatal physiology. Our serial measurements expand on this by demonstrating that a substantial portion of this maturation occurs within the first week. The observed increases in E/A and e′ reflect enhanced active relaxation, consistent with structural remodeling of the immature myocardium characterized by a gradual reduction in stiffness and improved calcium handling [17, 18]. Although diastolic function in our cohort had not yet reached term-equivalent levels by day 7, the trajectory suggests robust postnatal adaptation of LV filling dynamics during this critical transitional window.

Diastolic dysfunction in preterm infants remains poorly defined. While adult guidelines identify dysfunction based on impaired ventricular filling and elevated left atrial pressures [19], these features in preterm neonates, often reflect physiologic immaturity rather than true pathology. Early postnatal echocardiographic indices, such as E/A and e′, are frequently low due to reduced compliance and immature relaxation, but typically improve over time as the myocardium matures [15, 16]. Persistent abnormalities beyond the expected transitional period, particularly when associated with clinical signs like pulmonary edema or respiratory instability, may more accurately reflect true dysfunction.

No single parameter adequately defines diastolic dysfunction in this population. We advocate for a multi-modal assessment strategy that integrates pulsed-wave (PW) Doppler, tissue Doppler imaging (TDI), and left atrial (LA) strain. PW Doppler evaluates transmitral E and A velocities but is preload-sensitive and may be confounded by fusion at high heart rates. TDI measures e′ and a′ velocities to assess myocardial relaxation and provides E/e′ ratio as an estimate of filling pressures, which has been linked to outcomes such as bronchopulmonary dysplasia [5, 20]. LA strain, an emerging angle-independent technique, reflects LA reservoir function and compliance and may offer added sensitivity in detecting elevated filling pressures [21–23].

Prior studies have generally focused on a single modality: Sirc and Kozak et al. reported only PW Doppler parameters, while Bussmann et al. focused exclusively on TDI [8, 15]. In contrast, our study employed a multi-modal approach, combining complementary insights from Doppler, TDI, and volumetric indices. This comprehensive method enhances diagnostic precision and offers a more nuanced understanding of transitional cardiovascular physiology. Our approach aligns with ASE adult guidelines, which recommend using three or more indices to establish diastolic dysfunction [19].

Although defining dysfunction was not the primary aim of our study, our data provide important reference trends. The observed increases in E, e′, and E/A across the first week reflect expected physiologic maturation. Infants deviating from these trajectories, particularly those with elevated E/e′ or increased atrial volumes, may warrant further cardiovascular evaluation.

Diastolic function in preterm infants should be assessed using a comprehensive, multi-modal framework. Our study contributes foundational data on early postnatal diastolic trends and supports future efforts to refine neonatal-specific diagnostic algorithms that incorporate developmental physiology and clinical context.

## Impact of Patent Ductus Arteriosus on Diastolic Function

Our findings underscore the substantial impact of a large-volume PDA shunt on cardiac performance. Infants classified as having a hsPDA demonstrated elevated E/e′ ratios and increased LA volumes compared to those without. These parameters reflect higher LA pressure and impaired diastolic function, consistent with the burden of volume overload from a significant left-to-right shunt. This relationship supports the concept that a volume-loaded left ventricle endures greater diastolic strain. de Waal et al. (2019) similarly reported that both advancing postnatal age, and the presence of a PDA were associated with a greater risk of developing diastolic dysfunction in preterm infants [23]. Their retrospective analysis identified prolonged ductal patency as the most frequent contributor to elevated filling pressures. Our prospective findings reinforce this association: even by day 7, persistent ductal patency was linked to measurable diastolic abnormalities.

These observations highlight the importance of early recognition and management of hsPDA, as ongoing volume load can exceed the adaptive capacity of the immature myocardium. It is noteworthy, however, that not all infants with a hsPDA in our cohort exhibited overt diastolic impairment; an observation also described by de Waal et al. (2023) [23]. In that cohort, while PDA was a prominent risk factor, some preterm infants maintained normal diastolic indices despite the presence of a shunt. This heterogeneity suggests that additional factors such as shunt magnitude, myocardial maturation, and individual cardiovascular reserve, modulate the diastolic consequences of a hsPDA. These findings underscore the need for a nuanced, functionally guided approach: the mere presence of a PDA does not equate to diastolic dysfunction. Instead, comprehensive functional echocardiographic assessment including indices like E/e′ or left atrial strain may help identify infants experiencing true pathologic volume loading [24, 25].

It is important to acknowledge that a hsPDA may exert hemodynamic effects on both the left and right ventricles. Recent data, including that by Toyoshima et al. [26], have highlighted the potential for increased pulmonary blood flow secondary to left-to-right ductal shunting to result in volume loading not only of the left atrium and ventricle but also of the pulmonary circulation and right heart. This volume burden may, in turn, impact right ventricular (RV) compliance and diastolic performance during the early postnatal transition.

Although a comprehensive analysis of RV diastolic function was beyond the scope of the present study, we noted that infants with larger hsPDAs occasionally demonstrated trends suggestive of altered RV filling dynamics, including mildly elevated tricuspid E/e′ ratios and lower RV e′ velocities. These findings, while exploratory, are consistent with prior studies suggesting that the immature right ventricle may be particularly susceptible to preload variation and altered interventricular interactions in the setting of a hsPDA.

Incorporating a systematic assessment of right ventricular diastolic function in future studies may enhance our understanding of the global cardiac response to ductal shunting. The inclusion of right-sided indices such as tricuspid inflow velocities, RV tissue Doppler parameters, and right atrial strain may provide a more comprehensive evaluation of biventricular diastolic adaptation and inform more nuanced approaches to hemodynamic monitoring and PDA management in preterm infants.

Exploratory analysis of PFO characteristics revealed that infants with larger (≥ 2.5 mm) left-to-right shunting PFOs exhibited trends toward lower indexed LA volumes and E/e′ ratios compared to those with smaller PFOs, suggesting an offloading effect on the left atrium. When stratified by hsPDA status, this pattern was more evident among infants without a significant ductal shunt, whereas among those with hsPDA, the differences were attenuated—potentially reflecting the dominant influence of ductal volume load. Although our study was underpowered to detect statistical significance, these physiologic trends underscore the importance of considering PFO anatomy and its interaction with other shunts when interpreting diastolic markers. Notably, Kappico et al. reported that infants with pulmonary hemorrhage and a hsPDA had significantly smaller or restrictive PFOs, potentially limiting left atrial decompression and exacerbating pulmonary venous congestion [27].

Ultimately, these data emphasize that the capacity of the preterm heart to handle increased preload varies significantly. When diastolic performance is compromised, pulmonary venous congestion may develop, exacerbating respiratory distress. Conversely, infants with preserved diastolic function may tolerate a moderate PDA without major clinical instability. This variability has important implications for PDA treatment strategies, reinforcing the need to balance early closure versus expectant management based on the infant’s diastolic adaptation.

## Diastolic Function and Respiratory Status

Recent studies have increasingly underscored the association between impaired diastolic function and adverse respiratory outcomes in neonates, suggesting a potential influence on both short- and long-term pulmonary morbidity [9, 10]. In our cohort, the few infants who required invasive ventilation showed a trend toward lower LV e′ velocities and higher E/e′ ratios, consistent with impaired myocardial relaxation. However, our limited sample size constrained the ability to reach statistical significance. Despite this, our findings are in line with a growing body of evidence suggesting that diastolic function plays a critical role in the cardiorespiratory adaptation of preterm infants.

Bussmann et al. (2018) were among the first to report that preterm neonates requiring mechanical ventilation within the first 24 h had significantly lower LV e′ and a′ velocities compared to those managed with noninvasive support. In their large observational study, reduced early diastolic relaxation remained independently associated with the need for invasive ventilation, even after adjusting for gestational age and other clinical variables [10]. These findings imply that neonates with compromised diastolic function may have limited ability to accommodate postnatal circulatory shifts, leading to pulmonary congestion and respiratory decompensation.

The mechanistic explanation is physiologically sound: a stiff or poorly relaxing left ventricle may struggle to accommodate increasing pulmonary venous return, resulting in elevated left atrial pressures, pulmonary edema, and impaired gas exchange. Supporting this concept, de Waal et al. (2023) demonstrated that preterm infants with more severe early respiratory disease—particularly those with persistent or worsening symptoms—exhibited a higher prevalence of diastolic dysfunction on echocardiography. Their comprehensive evaluation, incorporating TDI, Doppler flow indices, and atrial strain analysis, showed that infants with unstable respiratory courses had significantly lower left atrial strain and impaired ventricular filling, while those with stable respiratory trajectories rarely exhibited signs of diastolic dysfunction [7].

Collectively, these findings reinforce that diastolic function is a key determinant in neonatal cardiorespiratory stability. The relationship is likely bidirectional: severe lung disease may precipitate myocardial strain through hypoxia, inflammation, or high intrathoracic pressures, while intrinsic myocardial dysfunction may predispose infants to respiratory deterioration by limiting effective preload accommodation. Notably, our cohort predominantly managed with noninvasive respiratory support offers a valuable lens through which to observe diastolic trends with minimal interference from mechanical ventilation. This strengthens the relevance of our findings by isolating intrinsic myocardial factors from iatrogenic effects.

In summary, our data and the existing literature point toward the importance of integrating cardiovascular assessment into the respiratory management of preterm infants. Early identification of diastolic dysfunction could help distinguish infants whose respiratory failure is exacerbated by cardiac compromise, enabling more tailored and proactive interventions.

## Strengths and Limitations

This study has several notable strengths. First, its prospective, longitudinal design allowed for serial echocardiographic assessments at defined time points within the same infants. This approach minimized inter-subject variability and provided valuable insight into the temporal evolution of diastolic function during the critical transitional period. Second, we employed a comprehensive assessment of cardiac function, evaluating both left and right ventricular diastolic parameters. This comprehensive evaluation provides a more complete picture of neonatal myocardial performance, particularly important given the relevance of RV function in the context of ventilatory pressures and ductal shunting. Third, our predominantly noninvasively ventilated cohort strengthens the applicability of our findings to modern neonatal practice. Most infants were managed with noninvasive support, limiting the confounding effects of sedation or high intrathoracic pressures from mechanical ventilation. Unlike prior studies that examined diastolic function later in neonatal life, our prospective serial design captures the rapid maturation within the first week, a period that had remained under-characterized.

Despite these strengths, several limitations should be acknowledged. The relatively small sample size limits statistical power to detect more subtle differences and constrains subgroup analyses, such as stratification by PDA status or type of respiratory support. Additionally, the broad gestational age range and the exclusion of approximately 15% of echocardiographic measurements due to technical limitations may introduce selection bias. Being a single-center study, our findings also reflect local management practices, including PDA treatment and ventilation strategies, which may limit generalizability. Multicenter studies would help validate the consistency of these findings in diverse NICU settings.

Technical challenges inherent to neonatal echocardiography further limit this work. TDI and transmitral inflow assessments in preterm infants are sensitive to probe angle and loading conditions, and image acquisition can be difficult in unstable or active neonates, particularly in the first 48 h. In addition, a formal intra-observer variability analysis was not completed for all cardiac cycles or parameters, primarily due to the technical demands of image acquisition and the time constraints of neonatal bedside echocardiography. These challenges are well recognized in the neonatal population, where limited acoustic windows, elevated heart rates, and motion artifacts often restrict optimal image quality. While reproducibility was assessed in the majority of studies and findings were qualitatively consistent, this remains a limitation. Finally, our analysis was limited to the first week of life, without follow-up beyond the neonatal period. As such, we cannot determine whether early diastolic abnormalities resolve or persist, nor can we comment on their potential predictive value for long-term outcomes such as bronchopulmonary dysplasia, growth, or neurodevelopment. Future longitudinal studies incorporating extended follow-up and correlation with clinical endpoints are warranted to better understand the significance of these early diastolic findings.

## Clinical Implications and Future Directions

Our findings carry important clinical implications for the care of VLBW infants. Early, serial assessment of diastolic function can serve as a valuable tool to detect cardiovascular compromise before overt clinical deterioration. Infants with poor relaxation or rising filling pressures may be at increased risk for respiratory instability or failure to wean from positive pressure support. Incorporating targeted neonatal echocardiography that includes diastolic indices may enhance risk stratification and support a more integrated approach to cardiopulmonary care [28].

Advances in echocardiographic techniques, such as speckle-tracking and left atrial strain imaging, offer more sensitive tools to assess diastolic function [21, 22]. These modalities may help distinguish between cardiac and pulmonary contributors to respiratory compromise and should be incorporated into future research.

Finally, larger prospective studies are needed to validate our findings and address key questions: How consistent are diastolic maturation patterns in larger and more diverse cohorts? Can early diastolic indices predict long-term outcomes like bronchopulmonary dysplasia or pulmonary hypertension? And most importantly, can interventions targeting diastolic dysfunction improve respiratory outcomes?

In summary, our study adds to the growing body of evidence that diastolic function plays a central role in neonatal cardiovascular stability. By delineating its early trajectory and its interplay with PDA and respiratory status, our findings offer valuable insight that may help guide more integrated strategies to support both cardiac and pulmonary health in preterm infants.

## Supplementary Information

Below is the link to the electronic supplementary material.

Supplementary file1 (DOCX 29 KB)

## Data Availability

The datasets generated and analyzed during the current study are not publicly available due to institutional policies and patient confidentiality restrictions, but are available from the corresponding author on reasonable request.
