# Supplementary material for: Evaluation of Biventricular Diastolic Function in Preterm Infants in the First Week of Postnatal Life
Source: Pediatr Cardiol. 2025 Jul 29;47(4):1818–28. doi: 10.1007/s00246-025-03974-7 (PMC12945902; doi:10.1007/s00246-025-03974-7)
Supplement: Supplementary file 1 — Supplementary file1 (DOCX 29 KB) [file 246_2025_3974_MOESM1_ESM.docx]

**Supplementary Table 2S. Diastolic Function and Cardiac Output Trends (Sensitivity Analysis Excluding Timepoints with Inotropic Support)**

| *Parameter* | *24 hours* | *48 hours* | *72 hours* | *day 7* |
| --- | --- | --- | --- | --- |
| *Mitral e/a ratio* | 0.90 [0.83–0.99] | 0.85 [0.78–0.91] | 0.89 [0.82–0.96] | 0.91 [0.84–0.98] |
| *Lateral e′ (cm/s)* | 3.4 [3.0–3.8] | 3.7 [3.2–4.1] | 3.8 [3.3–4.2] | 4.0 [3.5–4.5] |
| *Septal e′ (cm/s)* | 3.3 [2.9–3.6] | 3.5 [3.0–3.9] | 3.5 [3.1–3.9] | 3.6 [3.1–4.0] |
| *Mitral e/e′ ratio* | 15.9 [13.8–18.0] | 13.3 [11.5–15.2] | 14.1 [12.0–15.9] | 13.0 [11.2–14.9] |
| *Tricuspid e/a ratio* | 0.78 [0.69–0.87] | 0.85 [0.77–0.93] | 0.83 [0.74–0.91] | 0.88 [0.80–0.96] |
| *Tricuspid e′ (cm/s)* | 4.7 [4.2–5.2] | 4.9 [4.4–5.5] | 5.0 [4.5–5.6] | 5.1 [4.5–5.6] |
| *Indexed la volume (ml/kg)* | 0.87 [0.75–1.00] | 1.00 [0.88–1.12] | 1.04 [0.93–1.18] | 1.06 [0.94–1.20] |
| *Atrial filling fraction* | 0.55 [0.50–0.62] | 0.52 [0.47–0.58] | 0.51 [0.46–0.57] | 0.50 [0.44–0.56] |
| *LV output (ml/kg/min)* | 192 [179–211] | 275 [258–298] | 280 [258–305] | 276 [250–308] |
| *RV output (ml/kg/min)* | 204 [191–227] | 259 [236–284] | 264 [238–287] | 261 [234–289] |

*Table 2S.* *Trends in diastolic function and cardiac output during the first postnatal week after excluding timepoints with inotropic support. Median [IQR] values for mitral and tricuspid Doppler indices, myocardial velocities, left atrial volume, atrial filling fraction, and ventricular outputs are shown. This sensitivity analysis highlights consistent maturational patterns in diastolic function independent of inotropic influence.*
